# Mortality after hospital admission for heart failure: improvement over time, equally strong in women as in men

**DOI:** 10.1186/s12889-019-7934-3

**Published:** 2020-01-10

**Authors:** J. Buddeke, G. B. Valstar, I. van Dis, F. L. J. Visseren, F. H. Rutten, H. M. den Ruijter, I. Vaartjes, M. L. Bots, on behalf of the Queen of Hearts and RECONNECT investigators

**Affiliations:** 1 Julius Center for Health Sciences and Primary Care, University Medical Center Utrecht, Utrecht University, PO Box 85500, Utrecht, GA 3508 The Netherlands; 2 Dutch Heart Foundation, The Hague, The Netherlands; 3 Laboratory of Experimental Cardiology, University Medical Center Utrecht, Utrecht University, Utrecht, the Netherlands; 4 Department of Vascular Medicine, University Medical Center Utrecht, Utrecht University, Utrecht, The Netherlands

**Keywords:** Prognosis, Heart failure, Mortality, Trends, Sex, Age

## Abstract

**Background:**

To assess the trend in age- and sex-stratified mortality after hospitalization for heart failure (HF) in the Netherlands.

**Methods:**

Two nationwide cohorts of patients, hospitalized for new onset heart failure between 01.01.2000–31.12.2002 and between 01.01.2008–31.12.2010, were constructed by linkage of the Dutch Hospital Discharge Registry and the National Cause of Death registry. 30-day, 1-year and 5 -year overall and cause-specific mortality rates stratified by age and sex were assessed and compared over time.

**Results:**

We identified 40,230 men and 41,582 women. In both cohorts, men were on average younger than women (74–75 and 78–79 years, respectively) and more often had comorbid conditions (37 and 30%, respectively). In the 2008–10 cohort, mortality rates for men were 13, 32 and 64% for respectively 30-day, 1-year and 5-year mortality and 14, 33 and 66% for women. Mortality rates increased considerably with age similarly in men and women (e.g. from 10.5% in women aged 25–54 to 46.1% in those aged 85 and older after 1 year). Between the two time periods, mortality rates dropped across all ages, equally strong in women as in men. The 1-year absolute risk of death declined by 4.0% (from 36.1 to 32.1%) in men and 3.2% (from 36.2 to 33.0%) in women.

**Conclusions:**

Mortality after hospitalization for new onset HF remains high, however, both short-term and long-term survival is improving over time. This improvement was similar across all ages and equally strong in women as in men.

## Background

The burden of heart failure on Western societies is increasing and is projected to continue to do so in the future [1, 2]. Ageing, with its associated increase in comorbid conditions, is a driving force behind the emerging epidemic [3, 4], as is the considerably improved survival after an acute myocardial infarction [5]. Hospitalization for heart failure comes with a high risk of both short-term and long-term mortality [4]. The mortality risk increases with age, as has been shown in a variety of studies [6, 7]. Several studies indicated that the prognosis after hospitalization is worse for men than for women [4, 6, 7]. Others suggested that the sex difference seems to attenuate towards comparable mortality risks [7, 8]. Data on time trends in short-term and long-term survival, stratified for both age and sex, is sparse [1, 6], but needed to establish if sex differences matter in prognosis after heart failure hospitalization [9]. Therefore, we assessed contemporary age-, and sex-stratified overall and cause-specific short-term and long-term mortality after hospitalization using nationwide cohorts of patients hospitalized for new onset for heart failure in the Netherlands.

## Methods

### Registries and linkage procedure

Details of the registries and linkage procedures used to construct nationwide cohorts of patients hospitalized for the first time for heart failure have been previously described [10, 11]. Briefly, the data of the Dutch Hospital Discharge Register (HDR), the Dutch Population Register (PR), and the National Cause of Death Register were linked using a unique record identification number based on a combination of birth, sex and postal code (unique for 84% of the population). The PR was used to obtain data on demographic characteristics, HDR was used to identify patients with a hospital admission for heart failure, and cause of death statistics were used to obtain data on causes of death following admission for heart failure [11]. The PR became electronically available from 1995 onwards. Linkage of the registries is therefore possible from 1995 and onwards. For this study data was available from 1995 to 2015. All linkages and analyses were performed in agreement with the privacy legislation in the Netherlands and conforms with the principles outlined in the Declaration of Helsinki [12].

### Study population

A prospective cohort of patients with heart failure was built by selecting patients from the HDR with a primary admission for the following International Classification of Diseases (ICD) 9th revision codes for heart failure: 428.0, 428.1, 428.9, 402.01, 402.11 and 402.91. Those with a hospital admission for heart failure in the previous 5 years were excluded to ensure that the admissions for heart failure were, with a high probability, first new onset admissions. To investigate differences in mortality risk over time, two cohorts were created: one cohort containing information about patients admitted for heart failure between 1 Jan 2000 and 31 Dec 2002 (in short: the 2000–02 cohort) and one cohort containing information from patients admitted between 1 Jan 2008 and 31 Dec 2010 (in short: the 2008–10 cohort). For both cohorts, patients were additionally divided in isolated left-sided heart failure (ICD-9: 428.1) and other heart failure (ICD-9: 428.0, 428.9, 402.01, 402.11 and 402.91) to allow evaluation of the value of this ICD subdivision.

### Outcomes

The main outcomes were 30-day, 1-year and 5-year overall mortality. Follow-up was defined as time from hospital admission for heart failure to the day the patients died or the end of study period. Cause specific mortality is reported for cardiovascular mortality (separately for heart failure, ischemic heart disease, cerebrovascular disease, and other cardiovascular disease), cancer mortality (separately lung cancer) and respiratory mortality (separately chronic obstructive pulmonary disease (COPD)), and chronic kidney disease/renal failure mortality. All ICD codes used are mentioned in Appendix 1.

### Other characteristics

Demographic information comprises age, sex, and marital status. We determined the presence of comorbidity by the Charlson comorbidity index based on previous hospital admissions [13], which is considered a valid measure to estimate comorbidity in clinical research [14]. The mean Charlson comorbidity index was calculated as well as the proportion of patients that had an index score of 1 or more. Data on the duration of the hospital admission was available. No information was available on severity of heart failure at the time of admission, nor was data available to allow for differentiation between heart failure with preserved ejection fraction and reduced ejection fraction.

### Validation of heart failure discharge codes

The accuracy of the heart failure discharge codes were assessed in a dedicated validation study. For each precision digit of code ICD-9 code 428, 50 patients of the University Medical Center Utrecht were randomly selected and the medical records of these patients were manually checked for correct discharge ICD-9 code and discharge date. These codes were 428.0 (congestive heart failure, unspecified), 428.1 (left heart failure), and 428.2 (heart failure, unspecified).

### Data analysis

Baseline characteristics are presented as absolute numbers and percentages for both the 2000–02 and the 2008–10 cohorts. Secondly, we provided absolute numbers and percentages of all-cause mortality, cardiovascular mortality, cancer mortality, respiratory mortality and renal mortality of patients who died within 30-days, 1-year and 5-years after admission for heart failure in the recent cohort, and presented that by sex. Next, we estimated the 30-days, 1-year and 5-year mortality risk after first admission for heart failure in the 2000–02 and the 2008–10 cohorts and stratified these results by age and sex. Potential differences in mortality in sex and age groups were tested with logistic regression analyses. All analyses were adjusted for the Charlson Comorbidity Index (Table 3). To explore whether change in mortality over time was statistically different between men and women, we added an interaction term between sex and time and compared this model with the model without the interaction term using the likelihood ratio test. Then, we investigated whether a previous hospital admission for overall cardiovascular disease, acute myocardial infarction, or chronic pulmonary disease was associated with increased 30-day, 1-year and 5-year mortality in men and women using Cox proportional hazard models adjusted for age. Finally, we estimated the mortality risks of isolated left-sided heart failure (ICD-9: 428.1) and other heart failure (ICD-9: 428.0, 428.9, 402.01, 402.11 and 402.91), by age and sex. All analyses were performed using SPSS 22.0 (SPSS Inc., Chicago, IL, USA) and a *p*-value < 0.05 was considered statistically significant.

## Results

### Cohort study

We identified 38,848 patients (19,309 men and 19,539 women) with a first admission for heart failure in the earlier cohort 2000–02 and 42,964 patients (20,921 men and 22,043 women) in the recent cohort 2008–10 (Table 1). In both cohorts, women were on average 4 years older than men at the time of hospital admission for heart failure. Charlson comorbidity index was ≥1 in 37% of men and in 30% of women. Length of admission reduced with 2 days over time, from 8 to 6 days in men and from 9 to 7 days in women (Table 1). Of the patients in the 2008–2010 cohort who died within 30-days, 1-year and 5-years, the majority died from a cardiovascular cause (53–65%), with no clear differences between men and women. The proportion of cardiovascular mortality as cause of death decreased with increasing survival time, while the proportion of cancer to the overall mortality increased with survival time (Table 2).

**Table 1:** Characteristics of men and women with a hospital admission for heart failure in the periods between 2000 and 2002 and 2008–2010

	2000–02		2008–10		2000–02		2008–10	
	Men		Men		Women		Women	
	n	%	n	%	n	%	n	%
Total No. patients	19,309		20,921		19,539		22,043	
Mean age in years at admission (SD)	74 (11)		75 (11)		78 (11)		79 (11)	
Mean Charlson comorbidity index (SD)	0.7 (1.1)		0.7 (1.2)		0.5 (1.0)		0.5 (1.0)	
Charlson comorbidity index score ≥ 1	7119	37%	7711	37%	5875	30%	6497	30%
Myocardial infarction	2866	15%	2485	12%	1690	9%	1514	7%
Stroke	1053	5%	1294	6%	941	5%	1319	6%
Peripheral artery disease	1122	6%	1085	5%	541	3%	496	2%
Renal disease	496	3%	800	4%	346	2%	534	2%
Cancer	1219	6%	1839	9%	996	5%	1400	6%
Marital state								
Single	7572	39%	7988	38%	15,249	78%	16,338	74%
Married^a^	11,737	61%	12,933	62%	4290	22%	5705	26%
Median length of admission in days (IQR)	8 (9)		6 (8)		9 (10)		7 (10)	

Abbreviations: *SD* Standard Deviation; *IQR* Interquartile Range

^a^Married or registered partnership

**Table 2:** Characteristics and causes of death of patients who died after hospital admission for heart failure in the period between 2008 and 2010

	30-day mortality				1-year mortality				5-year mortality			
	Men		Women		Men		Women		Men		Women	
	n	%	n	%	n	%	n	%	n	%	n	%
Total deaths	2738	100%	3056	100%	6713	100%	7265	100%	13,351	100%	14,521	100%
Mean age at admission in years (SD)	81 (9)		84 (9)		80 (9)		83 (9)		78 (9)		82 (9)	
Median survival time in days (IQR)	8 (13)		7 (12)		52 (157)		49 (148)		360 (884)		365 (905)	
Cause of death												
Cardiovacular disease	1748	64%	1996	65%	3845	57%	4350	60%	7126	53%	8021	55%
Heart failure	563	21%	725	24%	1153	17%	1499	21%	2151	16%	2804	19%
Ischaemic heart disease	628	23%	516	17%	1302	19%	962	13%	2293	17%	1659	11%
Myocardial infarction	298	11%	275	9%	553	8%	481	7%	929	7%	811	6%
Cerebrovascular disease	52	2%	57	2%	144	2%	216	3%	361	3%	525	4%
Other cardiovascular disease	505	18%	695	23%	1246	19%	1673	23%	2321	17%	3033	21%
Cancer	240	9%	170	6%	867	13%	653	9%	1878	14%	1362	9%
Lung cancer	64	2%	30	1%	253	4%	116	2%	538	4%	245	2%
Respiratory disease	382	14%	427	14%	910	14%	834	12%	1758	13%	1669	12%
COPD	158	6%	182	6%	437	7%	380	5%	932	7%	799	6%
Chronic kidney disease/ Renal failure	31	1%	35	1%	114	2%	141	2%	259	2%	326	2%
Other cause	337	12%	428	14%	977	15%	1287	18%	2330	18%	3143	22%

Abbreviations: *COPD* Chronic Obstructive Pulmonary Disease

### Thirty-day mortality

**Fig. 1:**
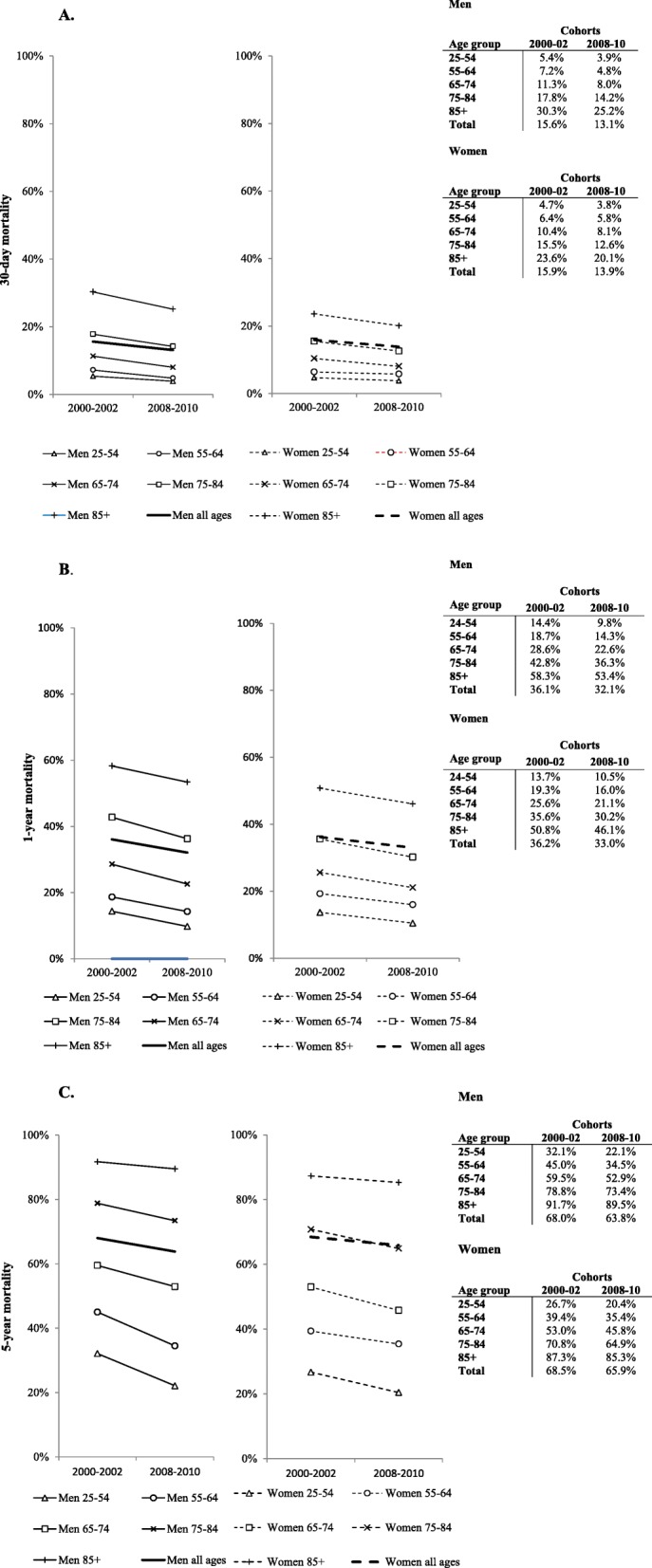
Trends in the 30-day (**a**), 1-year (**b**) and 5-year (**c**) mortality by sex and age

Short term mortality increased with age for both sexes (Fig. 1, Table 3A and B). Between 2000 and 02 and 2008–10, mortality after hospitalization for heart failure decreased in all age groups in both men and women. These decreases were statistically significant in men of most ages, except men aged 25–54, and in women aged 64 years or younger (Table 3A and B). Table 3C shows that the decline in mortality between the two time periods did not significantly differ between men and women.

**Table 3:** Difference in 30-day, 1-year, and 5-year mortality risk between the 2000–02 cohort and the 2008–10 cohort in men (A) and in women (B) and the difference in mortality risk between 2000 and 02 and 2008–10 between men and women (C)

	30-day mortality					1-year mortality				5-year mortality			
	Age group	2000–02	2008–10	OR^a^ (95% CI)	*p*	2000–02	2008–10	OR^a^ (95%CI)	*p*	2000–02	2008–10	OR^a^ (95%CI)	*p*
A	25–54 (%,n)	5.4% (60)	3.9% (45)	0.73 (0.49–1.08)	0.12	14.4% (160)	9.8% (114)	0.68 (0.52–0.88)	< 0.01	32.1% (357)	22.1% (256)	0.62 (0.51–0.75)	< 0.01
Men	55–64 (%,n)	7.2% (172)	4.8% (125)	0.65 (0.51–0.82)	< 0.01	18.7% (446)	14.3% (374)	0.73 (0.62–0.85)	< 0.01	45.0% (1075)	34.5% (901)	0.64 (0.57–0.72)	< 0.01
	65–74 (%,n)	11.3% (645)	8.0% (412)	0.68 (0.60–0.78)	< 0.01	28.6% (1629)	22.6% (1155)	0.75 (0.66–0.78)	< 0.01	59.5% (3384)	52.9% (2707)	0.76 (0.70–0.82)	< 0.01
	75–84 (%,n)	17.8% (1330)	14.2% (1125)	0.76 (0.70–0.83)	< 0.01	42.8% (3201)	36.3% (2888)	0.75 (0.71–0.80)	< 0.01	78.8% (5895)	73.4% (5833)	0.73 (0.67–0.78)	< 0.01
	85+ (%,n)	30.3% (800)	25.2% (1031)	0.79 (0.71–0.88)	< 0.01	58.3% (1539)	53.4% (2182)	0.82 (0.74–0.90)	< 0.01	91.7% (2421)	89.5% (3654)	0.75 (0.63–0.89)	< 0.01
	Total (%,n)	15.6% (3007)	13.1% (2743)	0.74 (0.70–0.79)	< 0.01^1^	36.1% (6975)	32.1% (6713)	0.75 (0.72–0.78)	< 0.01^1^	68.0% (13132)	63.8% (13351)	0.72 (0.69–0.76)	< 0.01^1^
B	25–54 (%,n)	4.7% (36)	3.8% (31)	0.81 (0.50–1.33)	0.38	13.7% (104)	10.5% (85)	0.77 (0.56–1.05)	0.10	26.7% (203)	20.4% (166)	0.72 (0.56–0.92)	< 0.01
Women	55–64 (%,n)	6.4% (76)	5.8% (84)	0.91 (0.66–1.25)	0.57	19.3% (231)	16.0% (231)	0.79 (0.65–0.98)	0.03	39.4% (470)	35.4% (512)	0.85 (0.72–0.99)	0.055
	65–74 (%,n)	10.4% (389)	8.1% (270)	0.76 (0.64–0.90)	< 0.01	25.6% (957)	21.1% (705)	0.78 (0.70–0.87)	< 0.01	53.0% (1981)	45.8% (1532)	0.75 (0.69–0.83)	< 0.01
	75–84 (%,n)	15.5% (1276)	12.6% (1055)	0.79 (0.72–0.86)	< 0.01	35.6% (2940)	30.2% (2534)	0.78 (0.73–0.83)	< 0.01	70.8% (5837)	64.9% (5447)	0.76 (0.71–0.81)	< 0.01
	85+ (%,n)	23.6% (1323)	20.1% (1616)	0.82 (0.75–0.89)	< 0.01	50.8% (2843)	46.1% (3710)	0.83 (0.77–0.89)	< 0.01	87.3% (4887)	85.3% (6864)	0.84 (0.76–0.93)	< 0.01
	Total (%,n)	15.9% (3100)	13.9% (3056)	0.79 (0.75–0.83)	< 0.01^1^	36.2% (7075)	33.0% (7465)	0.79 (0.76–0.83)	< 0.01^1^	68.5% (13378)	65.9% (14521)	0.78 (0.75–0.82)	< 0.01^1^
C	Differences men and women												
		Men∆2000–02 2008–10	Women∆2000–02 2008–10	OR^b^ (95%CI)	*p*	Men∆2000–02 2008–10	Women∆2000–02 2008–10	OR^b^ (95%CI)	*p*	Men ∆2000–02 2008–10	Women∆2000–02 2008–10	OR^b^ (95%CI)	*p*
	25–54	−1.5%	−0.9%	1.13 (0.60–2.11)	0.71	−4.6%	−3.2%	1.12 (0.75–1.68)	0.58	−10.0%	−6.3%	1.15 (0.84–1.57)	0.29
	55–64	−2.4%	−0.6%	1.40 (0.94–2.09)	0.10	−4.5%	−3.4%	1.10 (0.85–1.41)	0.48	−10.5%	−4.0%	1.32 (1.09–1.63)	< 0.01
	65–74	−3.3%	−2.3%	1.11 (0.90–1.36)	0.34	−5.1%	−4.5%	1.09 (0.94–1.25)	0.35	−6.6%	−7.2%	1.00 (0.89–1.13)	0.99
	75–84	−3.6%	−2.9%	1.03 (0.91–1.16)	0.67	−6.5%	−5.5%	1.03 (0.94–1.13)	0.48	−5.4%	− 5.1%	1.05 (0.95–1.16)	0.38
	85+	−5.1%	−3.6%	1.04 (0.90–1.19)	0.62	−4.9%	−4.7%	1.01 (0.90–1.14)	0.86	−2.2%	−2.0%	1.11 (0.91–1.34)	0.29
	Total	−2.5%	−2.0%	1.04 (0.97–1.13)	0.29^1^	−4.0%	−3.2%	1.05 (0.98–1.11)	0.16^1^	−4.2%	−2.6%	1.09 (1.02–1.15)	0.01^1^

^1^Adjusted for age

Abbreviations: CI confidence interval, OR Odds Ratio

^a^ Odds Ratio (OR) represents the odds of mortality after hospital admission for heart failure between 2008 and 2010 compared to 2000–2002 stratified by age and adjusted for Charlson comorbidity index

^b^ OR represents the odds of change in mortality (after hospital admission for heart failure between 2008 and 2010 compared to 2000–2002) in men compared to the same change in mortality in women stratified by age and adjusted for Charlson comorbidity index

### One-year mortality

One-year mortality also increased with age for both sexes (Fig. 1, Table 3A and B). Between 2000 and 02 and 2008–10, one-year mortality after hospitalization for heart failure decreased in all age groups in both men and women. These decreases were statistically significant in all men and women, except women aged 25-54 years (Table 3A and B). Table 3C shows that the decline in mortality between the two time periods did not significantly differ between men and women.

### Five-year mortality

Lastly five-year mortality increased with age for both sexes (Fig. 1, Table 3A and B). Between 2000 and 02 and 2008–10, five-year mortality after hospitalization for heart failure decreased in all age groups in both men and women. These decreases were statistically significant in men and women at all ages, except women aged 55-64 years (Table 3A and B). Table 3C shows that the decline in mortality between the two time periods was more pronounced in men compared to women when age groups were combined (decline men: 4.2% and women: 2.6%, *p*-value = 0.01) as well as in those aged between 55 to 64.

### Previous admission for cardiovascular disease

A previous hospital admission for cardiovascular disease yielded significantly lowered hazard ratio’s (HR) for 30-day mortality in both men and women and for 1-year mortality in women (Table 4). For example, a hospital admission for CVD any time in the 5 years preceding the hospital admission for heart failure was associated with a 24% lower risk of death within 30 days (HR: 0.76, 95% confidence interval (CI): 0.67–0.86)). In men, a hospital admission for chronic pulmonary disease 1 or 5 years before hospital admission for heart failure was associated with 28 and 29% lower risk of death within 30 days after hospitalization for heart failure. In women, a similar relation for a previous hospital admission for chronic pulmonary disease was observed only for death within 1 year after hospital admission for heart failure (Table 4).

**Table 4:** Previous admission for cardiovascular disease and the risk of death within 30 days, 1 year and 5 years after hospital admission for heart failure in the period between 2008 and 2010

	30-day mortality		1-year mortality		5-year mortality	
	Men	Women	Men	Women	Men	Women
Previous hospital admission for						
AMI: 30 days prior to HF admission	1.00 [0.69–1.43]	0.82 [0.51–1.30]	0.95 [0.82–1.10]	0.83 [0.66–1.05]	0.95 [0.82–1.10]	1.02 [0.88–1.21]
AMI: 1-year prior to HF admission	0.88 [0.70–1.11]	0.90 [0.67–1.21]	0.94 [0.86–1.02]	0.86 [0.74–1.01]	0.94 [0.86–1.02]	0.95 [0.86–1.06]
AMI: 5-year prior to HF admission	**0.78 [0.65–0.94]**	0.87 [0.69–1.09]	**0.93 [0.87–1.00]**	**0.77 [0.67–0.87]**	**0.93 [0.87–1.00]**	1.00 [0.91–1.10]
CVD: 30 days prior to HF admission	0.85 [0.62–1.15]	**0.70 [0.49–0.99]**	0.95 [0.83–1.08]	0.92 [0.75–1.12]	0.95 [0.83–1.08]	0.96 [0.84–1.10]
CVD: 1-year prior to HF admission	**0.75 [0.64–0.88]**	**0.79 [0.65–0.96]**	0.98 [0.92–1.05]	**0.88 [0.78–0.98]**	0.98 [0.92–1.05]	1.00 [0.92–1.07]
CVD: 5-year prior to HF admission	**0.76 [0.67–0.86]**	**0.79 [0.68–0.91]**	0.96 [0.92–1.01]	**0.81 [0.75–0.88]**	0.96 [0.92–1.01]	1.02 [0.96–1.08]
CPD: 30 days prior to HF admission	0.94 [0.63–1.39]	0.65 [0.42–1.00]	1.03 [0.88–1.20]	1.09 [0.83–1.43]	1.03 [0.88–1.20]	1.05 [0.87–1.27]
CPD: 1-year prior to HF admission	**0.72 [0.59–0.90]**	0.80 [0.63–1.01]	1.03 [0.94–1.12]	0.87 [0.76–1.01]	1.03 [0.94–1.12]	1.06 [0.96–1.17]
CPD: 5-year prior to HF admission	**0.71 [0.60–0.88]**	0.88 [0.69–1.14]	1.01 [0.92–1.10]	**0.85 [0.73–0.98]**	1.01 [0.92–1.10]	1.01 [0.91–1.11]

Multivariate Cox Regression model adjusted for age. Results are expressed as hazard ratios with 95% confidence intervals

Abbreviations: *AMI* Acute myocardial infarction; *CVD* Cardiovascular Disease (including AMI, Cerebrovascular Accident, Rheumatic Heart Disease and Peripheral Vascular Disease); *CPD* Chronic pulmonary disease

### Validation study

ICD codes were validated for 152 patients using the medical information registered in the electronic patient data system of the University Medical Center Utrecht (Table 5). 80% of these patients were correctly diagnosed with heart failure. However, this value varied across subcodes, from 76% for ICD-9 428.9 to 87% for ICD-9 428.0. In addition, 12% (428.0) to 22% (428.9) of the patients had heart failure as a complication during hospital stay. The remaining 2–6% did not have heart failure during hospital admission nor in their history, and may be considered misclassified. In figure Appendix 2 we explored the value of the ICD heart failure subcoding in terms of mortality risk. There were no differences in one-year mortality between those classified as isolated left-sided heart failure compared to the other codes in men, nor in women stratified for age. These results hold for 30-day and five-year mortality rates (results not shown).

**Table 5:** Results of validation of International classification of disease codes 428.0, 428.1 and 428.9

	428.0	428.1	428.9
Correct use of ICD code n, % (95% CI)	45, 87% (77–96%)	40, 80% (69–91%)	38, 76% (64–88%)
Incorrect use of ICD code n, % (95% CI)	1, 2% (0–6%)	3, 6% (0–13%)	1, 2% (0–6%)
Heart failure is complication during hospital stay n, % (95% CI)	6, 12% (3–21%)	7, 14% (4–24%)	11, 22% (11–33%)
Total (n)	52	50	50

Abbreviations: *CI* confidence interval, *ICD* International Classification for Disease

## Discussion

In this study we showed improvement in short-term and long-term survival after hospital admission for new onset heart failure hospitalizations between 2000 and 2010, although mortality rates are still high. This improvement was similar across all ages and equally strong in women as in men.

### Trends in survival

Our finding of a decline in mortality over this time period are in line with several other population based studies [1–9]. A similar decline in women as in men is in line with contemporary data suggesting that hospital care is similar for men and women with heart failure [15], and acute myocardial infarction [16]. These studies suggested that the decline is a result of better adherence, with no differences regarding sex, to optimized treatment as recommended in guidelines for heart failure. We confirm the steep increase in mortality after hospitalization for heart failure with increasing age, which also has been observed in a large number of previous studies [6, 7]. Interestingly, previous hospital admission for some conditions that may underlie the development of heart failure, e.g. cardiovascular disease, myocardial infarction and chronic pulmonary disease was associated with a reduced mortality risk of those admitted for new onset heart failure. Although surprising, this observation may be explained by the notion that patients with a known history of cardiovascular disease or respiratory disease may be referred to the hospital earlier compared to patients without known cardiovascular or respiratory disease. As a result, their stage of heart failure may be less advanced. Furthermore, due to the initiation of preventive cardiovascular medication, their cardiovascular condition may be better and thus their cardiovascular risk may be lower at the time of hospital admission when compared to a patient presenting with heart failure without a previous cardiovascular condition. This is however speculative and could not be investigated with the current data.

An in-depth explanation of observed trends in mortality with the current data is hampered by the fact that the database does not contain information on cardiovascular risk factors and medication use linked to the individuals. We know from previous work into coronary heart disease mortality trends that in the time window 1997–2007, on a population level systolic blood pressure fell, cholesterol level declined, and favorable changes occurred in smoking and physical activity [17]. These risk factor changes may have potentially led to an improved cardiovascular status at the time of heart failure hospitalisation leading to a reduction of risk afterwards. Furthermore, the uptake of beta-blockers in heart failure patients in the acute phase and for secondary prevention more than doubled between 1997 and 2007 [18]. Also the uptake of lipid lowering, blood pressure lowering drugs and beta blockers in the acute phase and secondary prevention phase of conditions predisposing to heart failure, such as acute coronary syndrome, may have favorably affected prognosis in the event that heart failure developed [18].

### Trends in survival stratified by sex

We confirm previous findings that showed significant decreases in mortality over time in both men and women and in all age groups [3, 19]. Heart failure with reduced ejection fraction (HFrEF) has been better recognized in the last decade and contemporary heart failure treatment largely improved mortality of patients with HFrEF, but not for those with preserved ejection fraction (HFpEF) [20]. Because men more often have HFrEF than women [2, 20], we expected to observe a more pronounced mortality decline in men similar to some previous studies [3, 19]. However, our data only show a somewhat more pronounced decline in five-year mortality for men, whereas no significant differences in decline for 30-day and one-year mortality were observed between men and women.

### Validity of ICD code

Validation of the ICD-9 heart failure codes yielded a high percentage of accuracy for the diagnosis of heart failure. Previous studies reported positive predictive values for the use of ICD-9 code 428 to identify patients with heart failure between 80% [21] and 94% [22]. This is in line with our estimate for 428 (80%) and supports the potential of using such data.

### Strengths and limitations

A strength of our study is the nationwide design with accordingly a large sample size, which enabled us to stratify our results for age and sex. Furthermore, the validity of the linkage of registries in the Netherlands has been proven to be high [21, 23–25]. Limitations of our study arise from the nature of hospital administrative data. Patients were identified on the basis of ICD-9 codes for heart failure. The ICD coding does not distinguish HFrEF and HFpEF. In addition, information on severity and prescribed medical treatment is not routinely collected in these registries, and thus more in depth analyses in causes underlying the observed trends is limited. Next, the Dutch HDR was electronically available from 1995. As we used data from 2000, the maximum wash-out-period to limit subsequent hospital admissions for heart failure was 5 years. As a result we may have included some patients with a recurrent admission for heart failure. The prognosis of these patients may be different from patients with a first admission for heart failure. The reported mortality rates may therefore in reality be somewhat lower or higher. However, we used a 5 years wash-out-period for both the 2000–02 cohort and the 2008–10 cohort and therefore it is not likely this has affected our trend estimates.

## Conclusions

In conclusion, mortality after hospitalization remains high, however, both short and long term survival is improving over time. This improvement was similar across all ages and equally strong in women and in men. These observational findings do not allow detailed evaluation of the underlying mechanisms.

## Figures and Tables

**Fig. 2:**
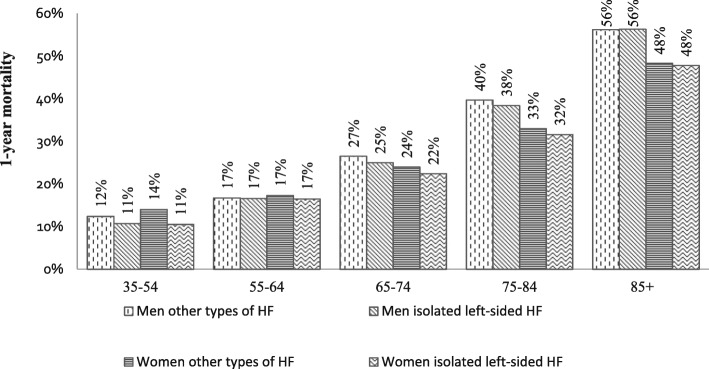
1-year mortality by heart failure type, sex and age group

## Data Availability

The data that support the findings of this study are available from the Central Bureau of Statistics Netherlands but restrictions apply to the availability of these data, which were used under license for the current study, and so are not publicly available. Data are however available from the authors upon reasonable request and with permission of the Central Bureau of Statistics Netherlands.

## Appendix 1

ICD-codes used in this study.

Hospital admissions were coded according to the International Classification of Diseases, Ninth Revision, Clinical Modification (ICD-9).

- Heart failure:
  428.0, 428.1, 428.9, 402.01, 402.11 and 402.91
- Left isolated heart failure:
  428.1
- Other heart failure:
  428.0, 428.9, 402.01, 402.11 and 402.91
- Acute myocardial infarction:
  410
- Chronic pulmonary disease:
  490-505, 506.4
- Cerebrovascular disease:
  430-438

Causes of death were coded according to ICD-10.

- Cardiovascular disease:
  D18, G45, I00-I99, K55, M30-M31, P29.3, Q20-Q28, R00-R01, R07.1-R07.4, R09.8, R23.0 and R59
- Heart failure:
  I50
- Ischaemic heart diseases:
  I20-I25
- Acute myocardial infarction:
  I21
- Cerebrovascular disease:
  I60-I69
- Cancer:
  C00-C97, D00-D48
- Lung cancer:
  C34
- Respiratory disease:
  J00-J99
- Chronic Obstructive Pulmonary Disease (COPD):
  J44
- Chronic kidney disease / Renal failure
  N17-N19

## Abbreviations

AMI: Acute myocardial infarction
CI: Confidence Interval
COPD: Chronic Obstructive Pulmonary Disease
CPD: Chronic pulmonary disease
CVD: Cardiovascular Disease
HDR: Hospital Discharge Register
HF: Heart Failure
HFpEF: Heart Failure with preserved Ejection Fraction
HFrEF: Heart Failure with reduced Ejection Fraction
HR: Hazard Ratio
ICD: International Classification of Disease
IQR: Interquartile Range
PR: Population Register
SD: Standard Deviation
